# The Cellient System for Paraffin Histology Can Be Combined with HPV Testing and Morphotyping the Vaginal Microbiome Thanks to BoonFixing

**DOI:** 10.1155/2013/502357

**Published:** 2013-02-20

**Authors:** Mathilde E. Boon

**Affiliations:** Leiden Cytology and Pathology Laboratory (LCPL), P.O. Box 16084, 2301 GB Leiden, The Netherlands

## Abstract

The Cellient Automated Cell Block System (Hologic) can be used to process cervical scrapes to paraffin sections. For the first study on this subject, cervical scrapes were fixed in the formalin-free fixative BoonFix. This pilot study was limited to cases classified as atypical squamous lesion of unknown significance (ASCUS) and high-grade squamous lesion (HSIL) as diagnosed in the ThinPrep slide. The Cellient paraffin sections were classified into negative, atypical, CIN 1, CIN 2, and CIN 3. Multiple HPV genotypes were encountered in 79% of the scrapes. This study showed that the Cellient system for paraffin sections can be combined with HPV testing thanks to the formalin-free BoonFix. In two additional studies it was shown that such samples can also be used for morphotyping the vaginal microbiome and preparing cytologic ThinPrep slides.

## 1. Introduction

### 1.1. The Cellient System

The Cellient Automated Cell Block System (Hologic), producing paraffin blocks could provide an ideal method for cytohistologists as it compares favorably with traditional cell block sectioning [1]. Although such automated techniques have an up front cost, they could prove to be cost-effective by saving preparation time for the cytologist, and thereby allowing more samples to be assessed in the required timeframe of clinical practice. In the paraffin sections, we can analyze p16 staining of dyskeratocytes [2] in cases classified as ASCUS (atypical squamous cells of unknown significance) and as HSIL (high-grade squamous intraepithelial lesion) [3].

## 2. Material and Methods

### 2.1. First Pilot Study of Combining Cellient Histology with HPV Testing

#### 2.1.1. Cervical Scrapes

In a pilot study of 14 HIV-positive women with ASCUS or HSIL, the Cellient system for cytohistology was exploited to analyze p16 positive dyskeratocytes in HPV-positive cervical scrapes [4]. Cervical scrapes of 14 HIV-positive women with a cytology diagnosis ASCUS or HSIL were selected for this first pilot study. A cervical scrape was taken as follows: the tip of the Cervex-Brush Combi (Rovers Medical Devices, Oss, the Netherlands) was placed in the endocervical canal and rotated. The Cervex-Brush Combi has the shape of a broom. These brooms were placed in a vial with the coagulant formalin-free fixative BoonFix, with polyethylene glycol (PEG) as one of its four components (Denteck, Zoetermeer, the Netherlands). Finally the vials with the samples were transported to the Leiden Cytology and Pathology Laboratory (LCPL) in Leiden, the Netherlands. In the Leiden laboratory each vial was placed in a commercial paint shaker and by its rigorous shaking all tissue fragments collected by the Cervex-Brush Combi emerged into the BoonFix solution.

#### 2.1.2. The ThinPrep Slides and Cytology Classification

From each sample six ThinPrep slides (Hologic) were prepared in the T3000. The remaining material in the vial was stored. The cytology ThinPrep slides were scored according to the Bethesda system [3].

#### 2.1.3. The HPV PCR Method

The BoonFixed samples were analyzed for 25 HPV genotypes using a highly sensitive PCR-reverse hybridization Line Probe Assay, the so-called INNO-LiPA HPV genotyping extra system (Innogenetics, Belgium). The SPF10 primer set amplifies a 65-bp region in the L1 open reading frame [5]. Detection after PCR is based on the principle of reverse hybridization. Amplification products are subsequently hybridized using specific oligonucleotide probes in a single typing strip [6]. For the current study, HPV-positive scrapes were included.

#### 2.1.4. The Cellient Paraffin Sections

All the remaining material in the vial was used for the Cellient method. This was collected into a cassette and loaded into the instrument. Eosin was applied and vacuum-drawn through the sample. Alcohol was similarly applied and drawn through the sample for dehydration. To clear the alcohol, the procedure was repeated with xylene. The sample was then embedded in paraffin and finally embedded in an additional layer of paraffin during processing in the finishing station.

From the Cellient paraffin block eight serial sections were cut, two for the Papanicolaou staining, one for the hematoxylin eosin (HE) staining, and five for the p16 immunostainings.

#### 2.1.5. Morphologic Criteria in ThinPrep and Cellient Slides

Metaplastic cells are defined as cells with a vesicular nucleus and a nuclear-cytoplasm ratio of around 0.5 [7]. In the p16 stain, the vesicular nature of the nucleus is still visible.

Koilocytotic cells display a clear zone well demarcated around the nucleus [7]. Particularly in the ThinPrep cytology slides it is easy to identify koilocytes.

Squamous pearls are whirls of squamous cells. In the ThinPrep slides these pearls display small pyknotic nuclei. The cytoplasmic staining is either turquoise or red. In the Papanicolaou-stained paraffin Cellient sections, the cytoplasm is either bright red or dark green; nuclei are not always in the section.

In the ThinPrep slides, dyskeratotic cells have a pyknotic nucleus, a nuclear cytoplasmic ration between 2 and 6, and orange or green staining cytoplasm. In the Papanicolaou-stained Cellient paraffin sections, the cytoplasm is green or orange-red. In these sections, the pyknotic nuclei of the dyskeratotic cells can be relatively large. In the HE stain, dyskeratotic cells have bright red cytoplasm. In the p16 Cellient paraffin sections, dyskeratotic cells can display brown cytoplasm and brown-blue nuclei or intensely brown pyknotic nuclei in which the blue (hematoxylin) staining is hidden by the brown color.

#### 2.1.6. BoonFix Is a Boon Fixative

The boon fixative BoonFix can be used for cervical scrapes [8] and for biopsies processed by microwave technology [9]. Note that the Cellient method is based on vacuum techniques and not on microwave technology as it is used on a large scale in the LCPL since 1987 [10, 11]. This fixative does not contain formalin nor methanol, the latter being an important component of Preserve of Hologic. In the Hologic system using Preserve, it is advised not to do PCR within one month of cervical sampling. In contrast, when the cervical scrape is suspended in BoonFix, PCR is possible on archival samples. In the studies presented herewith, Preserve from Hologic was not tested. To the best of our knowledge, no papers on this subject were published.

## 3. Results and Discussion

### 3.1. The HPV Genotypes and p16 Staining

In total, 11 HPV genotypes were found, two low risk (lr) (HPV44 and HPV70) and nine high risk (HPV16, HPV26, HPV51, HPV52, HPV53, HPV56, HPV58, HPV66, HPV69). In Table 1 low-risk and high-risk HPV genotypes and biomarker p16 staining are shown. All 14 scrapes were positive for hrHPV, and in addition scrapes 2, 4, 12, and 13 were positive for lrHPV. Multiple HPV genotypes were encountered in 11/14 (79%) cases.

In Table 2 the cytologic diagnoses of the Papanicolaou-stained ThinPrep slide classified as ASCUS and as HSIL and the histology diagnoses of the Papanicolaou- and HE-stained Cellient paraffin slides are presented. In three cases, metaplastic cells were observed in the paraffin Cellient sections, in seven cases koilocytosis, in one squamous pearls, and in seven dyskeratosis. As many as five cases (1, 2, 7, 11, and 14) had p16 positive dyskeratocytes. Only in case 3, 4, and 5 no p16 staining cells were observed in the Cellient paraffin sections.

#### 3.1.1. The Cellient Images

The Cellient Automated Cell Block System was able to provide high quality histology and outstanding images obtained with the biomarkers p16, all this on cervical scrapes on which 22 HPV genotypes were established by PCR.

Nuclear and cytoplasmic p16 positivity was observed in metaplastic cells, koilocytes, and dyskeratotic cells. Of the CIN 1 epithelial fragments, 10–30% of the cells were p16 positive, whilst in CIN 3 epithelial fragments over 50% stained positive. This is in accordance with our experience of p16 in Shandon cytoblock paraffin sections. Note that p16 positivity can also be observed in the cytohistology of adenocarcinoma of the endocervix [12].

It is well-known that HPV infection can induce a disturbance of the keratinization process leading to dyskeratosis. The Papanicolaou method (in which dye competition between light green and eosin is exploited) is well-suited to detect these dyskeratotic cells by their strikingly orange color, whilst others stain intensely green. It should be noted that the pyknotic nuclei of dyskeratotic cells can be rather large suggesting polyploidy. Such dyskeratotic cells can have relatively large nuclei and accordingly be designated as atypical. The biomarker p16 is used to detect overexpression of p16^INK4a^ protein in cervical premalignant and malignant lesions [13–15]. Galgano et al. [16] conclude that immunocytochemical staining for p16 is a useful and reliable adjunct for the identification of CIN 2+. We can add that in the Cellient paraffin sections of cervical scrapes, the p16 positive staining dyskeratotic cells can be identified and classified according to their nuclear size and nuclear cytoplasmic ratio.

### 3.2. HPV and the Vaginal Microbiome

The BoonFixed samples can in addition be used for morphotyping the vaginal microbiome. In a study of South African women, the vaginal microbiome was assessed by the Leiden BV scoring system based on morphotyping the stained bacteria. The cases were identified as Leiden bacterial vaginosis (BV) negative (BV−; *n* = 41), Leiden BV intermediate (BV±; *n* = 25), and Leiden BV positive (BV+; *n* = 34). These findings indicate a low BV– prevalence in these African women.

The 51 HIV positive women were frequently infected with HPV16 and HPV18. In addition, HPV35, HPV52, HPV33, and HPV66 were often detected in these samples. 

### 3.3. HPV Testing and Cytology Scoring of ThinPrep Slides

In BoonFixed cervical scrapes HPV testing can be combined with cytology on ThinPrep slides. The cross-sectional study of Dols et al. [17] was performed for 194 HIV-positive women. The cervical samples were genotyped for HPV types, and slides were evaluated for atypical squamous cell changes according to the Bethesda classification system. The cytology score of the ThinPrep slides versus lrHPV and hrHPV testing is presented in Tables 3 and 4. Prevalence of high grade squamous intraepithelial dysplasia (HSIL) was 9%. Overall, more than half (56%) of women were infected with an hrHPV type, 94% of women with HSIL (*n* = 16), 90% of women with LSIL (*n* = 35), and 42% of women within normal limits (WNL) (*n* = 58) tested positive for hrHPV. Overall, the most prevalent hrHPV subtypes were HPV16 (26%) and HPV52 (30%).

## 4. Conclusion

We conclude that the Cellient system for paraffin histology can be successfully combined with HPV testing and morphotyping the vaginal microbiome thanks to BoonFixing.

## Figures and Tables

**Table 1:** Low-risk and high-risk HPV genotypes and biomarker p16 staining.

Case	Low-risk HPV	High-risk HPV	p16 positive cells
1		52, 69	Dyskeratotic cells
2	70	52, 69	Dyskeratotic cells
3		51	No p16 staining
4	70	66	No p16 staining
5		51, 66	No p16 staining
6		16, 56	CIN 1 cells
7		16, 56	CIN 1 cells and dyskeratotic cells
8		16, 51, 53	Metaplastic cells
9		16, 53, 66	Koilocytotic cells
10		16, 53	Metaplastic cells
11		16	CIN 3 cells and dyskeratotic cells
12	44	26, 52	CIN 3 cells
13	70	52	CIN 3 cells
14		58	CIN 3 cells and dyskeratotic cells

**Table 2:** Cytology of the ThinPrep slide and cytohistology of the Cellient paraffin sections.

Case	Cytology	Cytohistology	Cell types
1	ASCUS	Atypia	Dyskeratosis and koilocytosis
2	ASCUS	Atypia	Dyskeratosis and koilocytosis
3	ASCUS	Atypia	Dyskeratosis and koilocytosis
4	ASCUS	Negative	Koilocytosis
5	ASCUS	Negative	Squamous pearls
6	ASCUS	CIN 1	CIN 1 fragments and dyskeratosis
7	ASCUS	CIN 1	CIN 1 fragments and metaplasia
8	ASCUS	Atypia	Dyskeratosis and metaplasia
9	ASCUS	Warty atypia	Koilocytosis
10	ASCUS	Negative	Metaplasia
11	HSIL	CIN 3	CIN 3 fragments and dyskeratosis
12	HSIL	CIN 3	CIN 3 fragments and koilocytosis
13	HSIL	CIN 3	CIN 3 fragments and koilocytosis
14	HSIL	CIN 3	CIN 3 fragments and dyskeratosis

**Table 3:** Cytology score of the ThinPrep slides versus HPV testing.

	Negative cytology (WNL)		ASCUS-LSIL		HSIL		Total	
	*n*	%	*n*	%	*n*	%	*n*	%
hrHPV positive	58	42.0	35	89.7	16	94.1	109	56.2
lrHPV positive	68	49.3	27	69.2	11	64.6	106	54.6
HPV negative	43	31.2	0		0		43	22.2

Total	138	71.1	39	20.1	17	8.8	194	100

**Table 4:** Cytology score versus hrHPV.

	Negative cytology (WNL)		ASCUS-LSIL		HSIL	
	*N*	%	*n*	%	*n*	%
hrHPV16	8	5.8	15	38.5	5	29.4
hrHPV18	6	4.3	1	2.6	2	11.8
hrHPV31	5	3.6	2	2.9	0	
hrHPV33	11	8.0	3	7.7	1	5.9
hrHPV35	6	4.3	3	7.7	4	23.5
hrHPV39	3	2.2	1	2.6	2	11.8
hrHPV45	1	0.7	0		0	
hrHPV51	10	7.2	3	7.7	2	11.8
hrHPV52	17	12.3	9	23.1	7	41.2
hrHPV56	3	2.2	3	7.7	0	
hrHPV58	3	2.2	8	20.5	0	
hrHPV59	0		1	2.6	0	
hrHPV66	7	5.1	13	33.3	2	11.8
hrHPV68	2	1.4	2	2.9	2	11.8

Total	138		39		17	
